# Whole-exome sequencing identifies a novel missense variant within *LOXHD1* causing rare hearing loss in a Chinese family

**DOI:** 10.1186/s12881-019-0758-2

**Published:** 2019-02-13

**Authors:** Na Shen, Ting Wang, Delei Li, Aiguo Liu, Yanjun Lu

**Affiliations:** 1 Department of Laboratory Medicine, Tongji Hospital, Tongji Medical College, Huazhong University of Science and Technology, Wuhan, 430030 China; 2 Department of Otorhinolaryngology, Tongji Hospital, Tongji Medical College, Huazhong University of Science and Technology, Wuhan, 430030 China

**Keywords:** Deafness, autosomal recessive 77 (DFNB77), Non-syndromic hearing loss (NSHL), *Lipoxygenase homology domains 1* (*LOXHD1*), Genetic variant, Whole-exome sequencing (WES)

## Abstract

**Background:**

Deafness, autosomal recessive 77 (DFNB77) is a rare non-syndromic hearing loss (NSHL) worldwide, which is caused by deleterious variants within *lipoxygenase homology domains 1* (*LOXHD1*). Here we identified that a novel missense variant of *LOXHD1* was associated with NSHL in a Chinese family under consanguineous marriage.

**Case presentation:**

A 28-year-old woman suffered a bilateral profound NSHL. Impedance audiometry, temporal bone computerized tomography (TBCT) scans and magnetic resonance imaging-inner ear hydrography (MRI-IEH) did not find any obvious abnormality of middle or inner ear. Routine genetic detection did not find pathogenic variants in common HL-associated genes. Therefore, we performed a whole-exome sequencing (WES) in this family. By trio-WES, co-segregation validation and bioinformatics analysis, we revealed that a novel homozygous variant in this patient, *LOXHD1*: c.5948C > T (p.S1983F), might be the pathogenic factor. Her parents (heterozygotes) and brother (wild-type) were asymptomatic.

**Conclusions:**

We successfully identified a novel variant of *LOXHD1* associated with a rare NSHL from a Chinese family. Our finds highlight the effectiveness of trio-WES for molecular diagnosis of rare NHSL, and expand the genotypic spectrum of DFNB77.

**Electronic supplementary material:**

The online version of this article (10.1186/s12881-019-0758-2) contains supplementary material, which is available to authorized users.

## Background

Hearing loss (HL) is the most common sensory deficit that affects 466 million people in the world (Available at http://www.who.int/pbd/deafness/estimates/en/). At least 60% of HL cases are accounted for genetic causes [1, 2]. Non-syndromic HL (NSHL) is the predominant type (~ 80%) of hereditary HL [3]. Nowadays, more than 100 genes have been related to NSHL (Available at https://hereditaryhearingloss.org/). However, except for several genes, many genes are insufficiently described due to low mutated frequencies, thus handicapping evidence-based genetic counseling on HL patients. Currently, the introduction of whole-exome sequencing (WES) makes it possible to screen all potential disease-causing genes for hereditary HL [4–6]. Benefiting from this technology, many HL patients could have molecular diagnosis when conventional methods identify no pathogenic variants within common HL-associated genes, thus helping to determine novel and more detailed genotype-phenotype correlations.

Deafness, autosomal recessive 77 (DFNB77, MIM # 613079) is a typical example of rare NSHL, which is caused by deleterious variants within *lipoxygenase homology domains 1* (*LOXHD1*) located at chromosome 18q21.1 (MIM #613072) [7]. LOXHD1 is a highly conserved stereociliary protein consisting of 15 polycystin-1/lipoxygenase/alpha-toxin (PLAT) domains, which facilitates proteins interacting with the plasma membrane [8]. *Loxhd1* in mice is mainly expressed in hair cell stereocilia and plays a crucial role in maintaining normal function of cochlear hair cells [7]. Mutations within *LOXHD1* are rare that only 33 pedigrees have been reported worldwide, harboring less than 50 different HL-causing variants to date [2, 7, 9–22]. Specially, these variants are extremely rare in East Asian population and only reported once in China [2, 14]. According to the HGMD database (http://www.hgmd.cf.ac.uk/docs/login.html, professional 2018.3 version), there are 47 missenses/nonsenses, 5 splicing variants, 5 small deletions, 1 small insertion, 1 small indel and 1 gross deletion identified in the *LOXHD1* gene. In these variants, 47 variants are associated with hearing loss and 16 variants are associated with late-onset Fuchs corneal dystrophy (FCD, MIM #136800). More studies are necessary to uncover potential genotype-phenotype correlations between *LOXHD1* variants and HL.

Here, we examined a Chinese family by trio-WES analyses and identified a novel missense variant, c.5948C > T (p.S1983F) within *LOXHD1* gene. This variant results in a bilateral NSHL.

### Case presentation

**Fig. 1:**
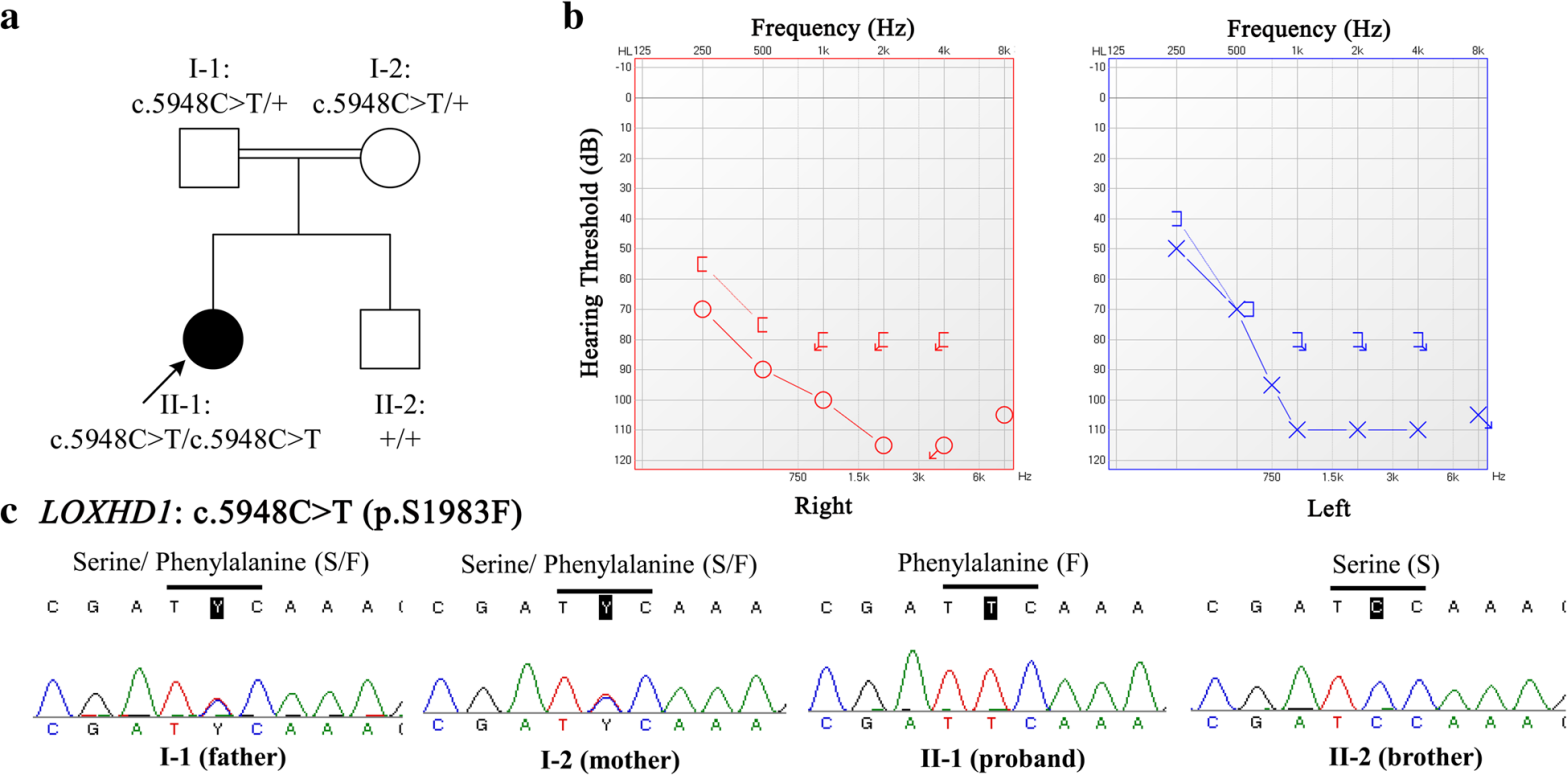
Pedigree, audiological evaluation and Sanger sequencing validation. **a**. Pedigree of this Chinese family under consanguineous marriage. The proband was indicated by arrows. “+” indicates wild type. **b**. Pure-tone audiometry evaluation of this proband. **c**. *LOXHD1*: c.5948C > T variants were validated by Sanger sequencing

The pedigree was shown in Fig. 1a. The proband (II-1) was a 28-year-old woman, who suffered a profound HL without any syndromic phenotype. She demonstrated a bilateral hearing loss at all frequencies and predominantly at middle to high frequencies, based on pure tone audiometry (PTA) test. The pure tone averages of 500 Hz, 1000 Hz and 2000 Hz were 97 dB HL in her both ears (see Fig. 1b). Impedance audiometry exhibited a typical A-type tympanogram for each ear. Temporal bone computerized tomography (TBCT) scans and magnetic resonance imaging-inner ear hydrography (MRI-IEH) did not find any obvious abnormality of middle or inner ear. Other associated symptoms were also not observed in the proband (II-1), including vestibular disorders (dizziness, vertigo, etc.), optic problems (blurred or distorted vision, eye pain, etc.), mal-development and intellectual disability. According to information provided by the family, II-1 had congenital HL but did not find obvious progression these years. No hearing or associated symptoms were found in the proband’s parents (I-1 and I-2) or brother (II-2). Her parents had consanguineous marriage. No deafness history was found in the last three generations of their family.

To identify the genetic cause of NSHL in this proband, we routinely applied a Sanger sequencing of four common HL-associated genes, including *gap junction protein beta-2* (*GJB2*), *gap junction protein beta-3* (*GJB3*), *solute carrier family 26 member 4* (*SLC26A4*) and *mitochondrially encoded 12S RNA* (*MT-RNR1*). DNA preparation, PCR conditions and Sanger sequencing process were described previously [23]. The coding regions of *GJB2* and *GJB3*, hotspot region (exon7–8 and exon19) of *SLC26A4*, and the full-length region of *MT-RNR1* were carefully screened, only a homozygous variant, m.827A > G within *MT-RNR1*, was identified. However, previous studies reported conflicting interpretations of pathogenicity for this variant [24–26], which was insufficient to result in hearing impairment.

Therefore, we further performed a WES analysis for the trio (I-1, I-2 and II-1) by using the Illumina HiSeq platforms. Details of the process of WES analysis are shown in Additional file 1: Supplementary materials. The target mean depths in the trio were all greater than 128X and > 97.8% of the target regions were covered by at least 20X. More than 77 thousands of variants were annotated for each person, and we summarize these results in Additional file 1: Table S1. Two analyses were applied in the trio data. One was de novo variants analysis, but we found no deleterious HL-associated variant. The other was shared variants analysis. A promising variant within *LOXHD1* (c. c.5948C > T; p.S1983F) was identified after rigorous filters (see Additional file 1: Tables S1 and S2). It was co-segregated and validated in this family by Sanger sequencing (see Fig. 1c). The primer sequences (5′ → 3′) were: forward-p, ATCGTGGTGCTTTTAACCTGC; reverse-p, GGGTGCTTGCACAGGATTG. Although homogeneous *MT-RNR1*: m.827A > G was identified in the proband, but her asymptomatic brother and mother also carrier this variant, implying that *MT-RNR1*: m.827A > G contributed little to the pathogenesis of the proband (Additional file 1: Table S3). *LOXHD1*: c.5948C > T was a missense variant, which was not found in all public databases (dbSNP, 1000 Genomes, ExAC and gnomAD), and predicted as damaging by multiple bioinformatics tools (SIFT, Polyphen2, and Mutation Taster, etc.). Evolution analysis also indicated that this variant was located at the well conserved region (Additional file 1: Table S2). Nowadays there have been 47 variants within *LOXHD1* associated with hearing impairment according to HGMD database, but c.5948C > T (p.S1983F) was not reported previously.

## Discussion and conclusions

NSHL is a complex disorder with highly genetic and clinical heterogeneity. Routinely, hotspot regions of four common HL-associated genes, such as *GJB2*, *GJB3*, *SLC26A4* and *MT-RNR1*, are recommended to be initially detected for molecular diagnosis for NSHL. If results are negative, gene-panel sequencing or WES are applied for further detection. Specially, a trio-WES is quite suitable for those rare NSHL. DFNB77 is a rare NSHL with autosomal recessive inheritance, caused by homozygous mutations within *LOXHD1* gene, firstly described in 2009 [7]. In the past ten years, about 60 variants within this gene were identified in NSHL cases. They showed different auditory characteristics, varying from stable to progressive and from mild to profound HL. The limited variant spectrum of *LOXHD1* strongly requires more studies to fill in gaps in the genotype-phenotype correlations of DFNB77. In this study, we used a trio-WES to successfully identify a novel homozygous variant, c.5948C > T (p.S1983F), within *LOXHD1* gene in a Chinese family. To the best of our knowledge, this is the second pedigree report of *LOXHD1*-related NSHL in China.

*LOXHD1* encodes an important protein consisting of 15 PLAT domains, which mediates protein interactions to maintain normal hair cell function [7]. Deleterious variants within *LOXHD1* could lead to various severities and various types of NSHL including progressive and non-progressive congenital HL [2, 7, 9–22]. Table 1 summarizes the published genotype-phenotype correlations of DFNB77 confirmed by segregation analysis. HL-associated variants within *LOXHD1* could occur in various races. Homozygotes (c.71delT/c.71delT, c.1588G > T/c.1588G > T, c.4212 + 1G > A/c.4212 + 1G > A, etc.) appeared to show a trend toward severe or profound HL, and compound heterozygotes showed different HL phenotypes. No overlapping genotype was reported by these studies performing segregation analysis. The quite limited information hindered to explore more genotype-phenotype correlations, requiring more studies to uncover variant spectrum of *LOXHD1* and related HL phenotype. Here, we identified a novel missense variant, *LOXHD1*: c.5948C > T, was associated with non-progressive NSHL in a family under consanguineous marriage. The proband carried homozygous c.5948C > T, her parents carried heterozygous c.5948C > T, and her brother did not carry this variant, which was compatible with the autosomal recessive inheritance of DFNB77. Comprehensive analyses, including family history, trio-WES, co-segregation validation, rarity in control population, and bioinformatics prediction, strongly support that *LOXHD1*: c.5948C > T could be a pathogenic factor. It makes effect on all the transcript isoforms of *LOXHD1* gene: NM_144612:exon38:c.5948C > T: p.S1983F, NM_001145473:exon7:c.851C > T:p.S284F, NM_001173129:exon7:c.851C > T:p.S284F, NM_001308013:exon19:c.2513C > T:p.S838F, and NM_001145472:exon21:c.2801C > T:p.S934F. Variants within *LOXHD1* are quite rare and recently, Hu et al. reported a first affected Chinese pedigree with progressive NSHL [2]. Compared to the compound heterozygotes (c.1751C > T/c.5815G > A) found by Hu et al., we identified a novel homozygote, c.5948C > T/ c.5948C > T, was associated with non-progressive NSHL. In addition, c.5948C > T is located in the 14th PLAT domain of LOXHD1 protein, which harbors the most published variants to date, compared with other PLAT domains [20]. Another five published variants (c.5869G > T, c.5885C > T, c.5934C > T, c.5944C > T and c.6162_6164delCCT) from different races are also concentrated in here [14, 18, 20], indicating that the 14th PLAT domain could be a hotspot mutated region of *LOXHD*1.

**Table 1:** Genotype-phenotype correlation of DFNB77 confirmed by segregation analysis

Genotype	Ethnicity	Severity of HL	Progression of HL	Reference
c.71delT/c.71delT	Turkish	Severe or profound	NA	[13]
c.442A > T/c.4217C > T	NA	NA	NA	[19]
c.1588G > T/c.1588G > T	Qatary	Severe to profound	Progressive	[12]
c.1618dup/c.1730 T > G	Dutch	Moderate to severe	Stable to progressive	[20]
c.1751C > T/c.5815G > A	Chinese	Severe	Progressive	[2]
c.1828G > T/c.2641G > A	Dutch	Mild	Stable	[20]
c.1904 T > C/c.4678 T > C	Dutch	Mild	Stable to progressive	[20]
c.2008C > T/c.2008C > T	Iranian	Mild to profound	Progressive	[7]
c.2696G > C/c.3834G > C	Dutch	Moderate	Stable	[20]
c.2696G > C/c.5934C > T	Dutch	Mild	NA	[20]
c.2863G > T/c.2863G > T	Turkish	NA	NA	[10]
c.3061C > T/c.5885C > T	Indian	Severe	Stable	[20]
c.3061 + 1G > A/c.6353G > A	Dutch	Moderate	NA	[20]
c.3076G > T/c.4375 + 1G > T	Japanese	Profound	Stable	[17]
c.3169C > T/c.6353G > A	Dutch	Severe	Stable	[20]
c.3371G > A/c.3979 T > A	Cameroonian	Profound	NA	[15]
c.3748 + 1G > C/c.6353G > A	Dutch	Moderate to severe	Stable to progressive	[20]
c.4212 + 1G > A/c.4212 + 1G > A	Japanese	Profound	Stable	[14]
c.4212 + 1G > A/c.5674G > T	Japanese	Mild to profound	Progressive	[16]
c.4480C > T/c.4480C > T	Turkish	NA	NA	[10]
c.4480C > T/c.5869G > T	Japanese	Moderate to severe	Stable	[14]
c.4623C > G/c.5545G > A	Czech	Severe	NA	[22]
c.4714C > T/c.4714C > T	Ashkenazi Jewish	Severe to profound	NA	[9]
c.5894dupG/c.5894dupG	Arab	Profound	NA	[21]
c.5948C > T/c.5948C > T	Chinese	Profound	Stable	This study

Abbreviation: *NA* not available

Variants within *LOXHD1* were also linked to late-onset FCD, a genetic degenerative disease of corneal endothelium towards blindness. In 2012, Riazuddin, et al. first reported a heterozygous damaging variant within *LOXHD1* in a multiplex family with dominant-inherited late-onset FCD [27]. However, subsequent studies failed to provide a strong association between *LOXHD1* variants and FCD [20, 28–30]. Specially, results from a Chinese multi-generational FCD pedigree demonstrated that no pathogenic variants were identified in *LOXHD1* [28]. In line with these previous studies, our work also did not observed any symptoms of FCD in the proband and her blood relatives within three generations. However, a limitation of our study is that the identified missense variant is lacking in animal models or in the verification of other HL patients. More functional and population studies are required to further verify our results.

In summary, we demonstrated that a novel missense variant, *LOXHD1*: c.5948C > T, was associated with non-progressive NSHL in a Chinese family under consanguineous marriage. Our work highlights the effectiveness of trio-WES for molecular diagnosis of rare NHSL and expands the variant spectrum of *LOXHD1* in hearing impairment.

## Additional file

Additional file 1: Supplementary Materials and Tables. (a) The process of whole-exome sequencing (WES) analysis. (b) **Table S1.** Filtering process of WES analysis in our study. (c) **Table S2.** Candidate gene and variant identified by trio-WES. (d) **Table S3.** Variants validated by Sanger sequencing. (DOC 59 kb)

## Abbreviations

DFNB77: Deafness, autosomal recessive 77
FCD: Fuchs corneal dystrophy
*GJB2*: *Gap junction protein beta-2*
*GJB3*: *Gap junction protein beta-3*
HL: Hearing loss
*LOXHD1*: *Lipoxygenase homology domains 1 l*
*MT-RNR1*: *Mitochondrially encoded 12S RNA*
NSHL: Non-syndromic hearing loss
*SLC26A4*: *Solute carrier family 26 member 4*
WES: Whole-exome sequencing
