# Supplementary material for: Whole-exome sequencing identifies a novel missense variant within LOXHD1 causing rare hearing loss in a Chinese family
Source: BMC Med Genet. 2019 Feb 13;20:30. doi: 10.1186/s12881-019-0758-2 (PMC6373029; doi:10.1186/s12881-019-0758-2)
Supplement: Supplementary file 1 — Supplementary Materials and Tables. (a) The process of whole-exome sequencing (WES) analysis. (b) Table S1. Filtering process of WES analysis in our study. (c) Table S2. Candidate gene and variant identified by trio-WES. (d) Table S3. Variants validated by Sanger sequencing. (DOC 59 kb) [file 12881_2019_758_MOESM1_ESM.doc]

**Supplementary Materials and Tables**

**The process of whole-exome sequencing (WES) analysis**

The WES was performed by the Illumina HiSeq X10 platform (San Diego, CA, USA). Frist, raw data was evaluated by fastp, then filtered clean reads was aligned to the reference genome (UCSC hg19, 2009) using the BWA-MEM, and followed by excluding PCR duplicates using SAMBLASTER and correcting reads using GATK. Second, variants including single-nucleotide polymorphisms (SNPs) and indels were identified by SAMtools and VarScan 2, and further annotated by ANNOVAR [1-5]. A promising candidate gene was considered that a variant met all the following criteria: (1) nonsense, missense, frame shift, or splice site variants; (2) absent in the repeat region; (2) absent or frequency < 1% in all the population databases, including dbSNP, gnomAD, ExAC, 1000genomes and ESP; (3) predicted as “pathogenic or damaging” in at least one softwares, such as SIFT, Polyphen2 and MutationTaster; and (4) novo variants (heterozygous variants in the proband that absent in her parents) or shared variants (homozygous variants in the proband that heterozygous in her parents).

**References**

1. Li H, Durbin R: **Fast and accurate short read alignment with Burrows-Wheeler transform**. *Bioinformatics (Oxford, England)* 2009, **25**(14):1754-1760.

2. Li H, Handsaker B, Wysoker A, Fennell T, Ruan J, Homer N, Marth G, Abecasis G, Durbin R: **The Sequence Alignment/Map format and SAMtools**. *Bioinformatics (Oxford, England)* 2009, **25**(16):2078-2079.

3. Wang K, Li M, Hakonarson H: **ANNOVAR: functional annotation of genetic variants from high-throughput sequencing data**. *Nucleic acids research* 2010, **38**(16):e164.

4. Koboldt DC, Zhang Q, Larson DE, Shen D, McLellan MD, Lin L, Miller CA, Mardis ER, Ding L, Wilson RK: **VarScan 2: somatic mutation and copy number alteration discovery in cancer by exome sequencing**. *Genome research* 2012, **22**(3):568-576.

5. Faust GG, Hall IM: **SAMBLASTER: fast duplicate marking and structural variant read extraction**. *Bioinformatics (Oxford, England)* 2014, **30**(17):2503-2505.

**Table S1. Filtering process of WES analysis in our study**

| Items | II-1 (proband) | I-1 (father) | I-2 (mother) |
| --- | --- | --- | --- |
| Target size (bp) | 39,501,604 | 39,501,604 | 39,501,604 |
| Coverage rate (%) | 99.68 | 99.85 | 99.70 |
| Target mean depth (X) | 128.97 | 166.54 | 136.94 |
| 20X coverage rate (%) | 97.84 | 98.75 | 98.16 |
| Total snp (filter offtarget) | 77,330 | 78,402 | 81,267 |
| exonic | 22,497 | 22,727 | 22,967 |
| splicing | 717 | 740 | 736 |
| nonsynonymous | 10,636 | 10,601 | 10,816 |
| stopgain | 91 | 95 | 94 |
| stoploss | 9 | 8 | 11 |
| Analysis of de novo variants in II-1 | 3,330 | 0 | 0 |
| De novo variants with high priority^*^ (A) | 49 | 0 | 0 |
| De novo variants within HL-associated genes (B) | 38 | 0 | 0 |
| Variants meeting the condition of A+B | 0 | 0 | 0 |
| Analysis of shared variants | 3,737 | 3,737 | 3,737 |
| Shared variants with high priority^*^ (C) | 13 | 13 | 13 |
| Shared variants within HL-associated genes (D) | 40 | 40 | 40 |
| Variants meeting the condition of C+D | 1 | 1 | 1 |
| Sanger confirmation in pedigree/Co-segregation | 1 | 1 | 1 |
| Causative gene and variant | *LOXHD1:*NM_144612: c.5948C>T | | |

^*^ Variants meeting all the following filters were considered as high priority: (1) not located in the repeated regions; (2) nonsynonymous or splicing variants; (3) minor allele frequency (MAF) < 0.01 in East Asian population from all databases including 1000 Genomes, ExAC and gnomAD; and (4) predicted as damaging by more than one bioinformatics tool, such as SIFT, Polyphen2 and MutationTaster.

Table S2. Candidate gene and variant identified by trio-WES

| Individual | Gene | Sex | Change of nucleotide and amino acid | Allele frequency in control population (dbSNP/gnomAD/ExAC/1000genomes/ESP) | Function prediction | Conservation |
| --- | --- | --- | --- | --- | --- | --- |
| II-1 (proband) | *LOXHD1* | Female | NM_144612: exon38 :c.5948C>T: p.S1983F | Not available in all above databases | SIFT (Damaging, score: 0.001); Polyphen2 (Probably damaging, score: 0.998); MutationTaster (Disease causing, score: 1); PROVEAN (Damaging, score: -3.54);VEST3 (Damaging, score: 0.912); CADD (Damaging, score: 33); GenoCanyon (Damaging, score: 1); FATHMM_MKL (Damaging, score: 0.992). | GERP (Conserved, score: 5.6); phyloP (Conserved, score: 9.219); phastCons (Conserved, score: 1); SiPhy (Conserved, score: 19.986). |

Table S3. Variants validated by Sanger sequencing

| Individual | Age (years) | Phenotype | Gene | Variants | Carrier status |
| --- | --- | --- | --- | --- | --- |
| II-1 (proband) | 28 | NHSL | *MT-RNR1* | m.827A>G | Homogeneous |
|  |  |  | *LOXHD1* | c.5948C>T | Homozygous |
| II-2 (brother) | 25 | Asymptomatic | *MT-RNR1* | m.827A>G | Homogeneous |
|  |  |  | *LOXHD1* | c.5948C>T | Wild type |
| I-1 (father) | 56 | Asymptomatic | *MT-RNR1* | m.827A>G | Wild type |
|  |  |  | *LOXHD1* | c.5948C>T | Heterozygous |
| I-2 (mother) | 53 | Asymptomatic | *MT-RNR1* | m.827A>G | Homogeneous |
|  |  |  | *LOXHD1* | c.5948C>T | Heterozygous |
